# Skeletal loading regulates breast cancer-associated osteolysis in a loading intensity-dependent fashion

**DOI:** 10.1038/s41413-020-0083-6

**Published:** 2020-02-14

**Authors:** Yao Fan, Aydin Jalali, Andy Chen, Xinyu Zhao, Shengzhi Liu, Meghana Teli, Yunxia Guo, Fangjia Li, Junrui Li, Amanda Siegel, Lianxiang Yang, Jing Liu, Sungsoo Na, Mangilal Agarwal, Alexander G. Robling, Harikrishna Nakshatri, Bai-Yan Li, Hiroki Yokota

**Affiliations:** 1 Department of Pharmacology, School of Pharmacy, Harbin Medical University, Harbin, 150081 China; 2 Department of Biomedical Engineering, Indiana University Purdue University Indianapolis, Indianapolis, IN 46202 USA; 3 Peking Union Medical College Hospital, Chinese Academy of Medical Sciences, Beijing, 100730 China; 4 Department of Physics, Indiana University Purdue University Indianapolis, Indianapolis, IN 46202 USA; 5 Department of Mechanical Engineering, Oakland University, Rochester, MI 48309 USA; 6 Integrative Nanosystems Development Institute, Indiana University Purdue University Indianapolis, Indianapolis, IN 46202 USA; 7 Department of Anatomy and Cell Biology, Indiana University School of Medicine, Indianapolis, IN 46202 USA; 8 Indiana Center for Musculoskeletal Health, Indiana University School of Medicine, Indianapolis, IN 46202 USA; 9 Department of Surgery, Simon Cancer Research Center, Indiana University School of Medicine, Indianapolis, IN 46202 USA

**Keywords:** Bone cancer, Bone cancer

## Abstract

Osteocytes are mechanosensitive bone cells, but little is known about their effects on tumor cells in response to mechanical stimulation. We treated breast cancer cells with osteocyte-derived conditioned medium (CM) and fluid flow-treated conditioned medium (FFCM) with 0.25 Pa and 1 Pa shear stress. Notably, CM and FFCM at 0.25 Pa induced the mesenchymal-to-epithelial transition (MET), but FFCM at 1 Pa induced the epithelial-to-mesenchymal transition (EMT). This suggested that the effects of fluid flow on conditioned media depend on flow intensity. Fluorescence resonance energy transfer (FRET)-based evaluation of Src activity and vinculin molecular force showed that osteopontin was involved in EMT and MET switching. A mouse model of tumor-induced osteolysis was tested using dynamic tibia loadings of 1, 2, and 5 N. The low 1 N loading suppressed tumor-induced osteolysis, but this beneficial effect was lost and reversed with loads at 2 and 5 N, respectively. Changing the loading intensities in vivo also led to changes in serum TGFβ levels and the composition of tumor-associated volatile organic compounds in the urine. Collectively, this study demonstrated the critical role of intensity-dependent mechanotransduction and osteopontin in tumor-osteocyte communication, indicating that a biophysical factor can tangibly alter the behaviors of tumor cells in the bone microenvironment.

## Introduction

Bone is a mechanosensitive organ, and it constantly remodels itself using mechanical loading as one of its major cues.^1^ Osteocytes are the most abundant type of cells in the bone matrix, and they act as mechanosensors and induce load-driven bone remodeling.^2^ While mechanical loading activates many load-sensitive genes via a wide spectrum of signaling pathways, little is known about the potential effects of mechanical stimulation on tumor-osteocyte communication. Osteocyte-mediated loading effects are potentially different from those observed in tumor cells that directly receive mechanical stimulation.^3–5^ In this study, we examined the interactions between osteocytes and migratory breast cancer cells in the presence and absence of mechanical stimulation in vitro and in vivo.

Bone is the most frequent site of metastasis of breast cancer.^6^ Interactions between tumor cells and bone-resorbing osteoclasts are known to induce a vicious feed-forward cycle in which osteolytic responses are amplified by a loop of interactions among PTHrP and TGFβ.^7^ In addition to interactions with osteoclasts, tumor cells are also attracted by chemoattractants in the bone marrow and ECM,^8^ and some ECM proteins, such as collagen and proteoglycans, are reported to act as attractants.^9^ For instance, type I collagen is the most abundant protein in the ECM of the bone matrix, and receptors such as integrin and discoidin domain proteins have been shown to interact with collagen and alter the expression of genes that are involved in the epithelial-to-mesenchymal transition (EMT).^10,11^ The major aim of this study was to identify the effect of mechanical stimulation on tumor-osteocyte communication.

We used conditioned media derived from osteocytes with and without mechanical stimulation and monolayer and 3D culture assays to evaluate whether mechanical stimulation induces differential effects on migratory breast cancer cells regarding their viability, migration, and EMT-linked gene expression. Furthermore, in a mouse model of breast cancer-associated osteolysis, we evaluated whether skeletal loading of tumor-loaded osteolytic bone inhibits or promotes tumor progression. In tumor-metastasized bone, tumor cells and osteocytes are considered the seed and soil. The chief focus in this study was the effect of mechanical stimulation on the soil (osteocytes and bone) and the subsequent impact of the modified soil on the seed (tumor-inoculated bone). Fluid flow-driven shear stress (0.25 Pa and 1 Pa) was applied to osteocytes, and tibia loading (1–5 N) was applied to tumor-inoculated bone.

In biophysical therapy to suppress inflammation and tissue degradation in osteoarthritic joints, we and others observed that low-intensity loads of the knee (0.5–1 N) provided beneficial outcomes, but medium loads (2 N or above) promoted the expression and activity of matrix metalloproteinases (MMPs),^12^ and excessive mechanical loading was shown to promote osteoarthritis.^13^ Based on these observations, our hypothesis is that medium mechanical stimulation in the form of oscillatory fluid flow (1 Pa shear stress) or skeletal loading (5 N loads) induces EMT-like responses in tumor cells, leading to increased migration and invasiveness. In contrast, we hypothesize that low mechanical stimulation (0.25 Pa shear stress or 1 N loads) reverses this effect and induces MET-like responses.

In this study, we used the breast cancer cell line MDA-MB-231 and its TMD cell (mouse xenografted clone) variant, human primary breast cancer cells,^14^ and the mouse mammary tumor cell lines 4T1, 4T1.2, and EO771. To induce mechanical stimulation in vitro, oscillatory fluid flow between a pair of parallel plates was applied to MLO-A5 osteocytes^15^ at 1 Hz with a peak shear stress of 0.25 Pa or 1 Pa. Tumor cells were then treated with MLO-A5-derived conditioned medium (CM) or MLO-A5 fluid flow-treated conditioned medium (FFCM). Using a mouse model of tibial osteolysis, tibia loading (oscillatory compressive loading) was applied to two mouse strains, C57BL/6 and BALB/c, after tibial injection of EO771 and 4T1.2 mouse mammary tumor cells, respectively. The daily loading condition was sinusoidal loads at 2 Hz with peak-to-peak loads at 1 N, 2 N, and 5 N for 5 min.

To analyze the EMT, we examined the expression of Snail and EMT-linked genes. To measure the activation of tumor-induced osteoclastic bone resorption, TGFβ, an ECM-enriched growth factor, was measured.^16^ The expression and activity of Src is known to correlate with advanced malignancy and poor prognosis in a variety of cancers.^17,18^ Osteopontin (OPN) is known to play a role in cell adhesion and migration and tumorigenesis.^19^ Mass spectrometry-based protein analysis predicted the involvement of OPN in the mechanosensitive migratory behaviors of tumor cells. OPN involvement in osteocyte-tumor interactions was further probed by analyzing the action of OPN on Src activity and molecular tension force using two fluorescence resonance energy transfer (FRET)-based techniques, as well as RNA interference. In a FRET experiment using a Src biosensor, the activity level of Src in response to varying amounts of OPN was evaluated based on the conformational change in the Src biosensor.^20^ In a second FRET experiment using a vinculin force biosensor, the migratory behavior of individual tumor cells was evaluated in which the FRET lifetime increased by stretching a molecular linker in the force sensor.^21^

The response to mechanical stimulation was further measured using proteins and volatile organic compounds (VOCs) that were designed to determine general levels of osteolysis and tumor progression. More specifically, we analyzed the expression of PPARγ, a transcription factor that promotes lipid metabolism and is generally associated with the pathogenesis and development of tumors.^22^ Cancer-associated VOCs are a generalized biomarker of disease.^23^ We therefore also measured VOC levels in the urine of differentially treated mice. The results of this study indicate that tumor-osteocyte interactions are affected by mechanical stimulation in vitro and in vivo, suggesting an important role for biophysical signals on metastatic tumor cells in bone.

## Results

### Differential effects of A5 osteocyte conditioned medium (CM) and fluid flow-treated CM (FFCM)

**Fig. 1:**
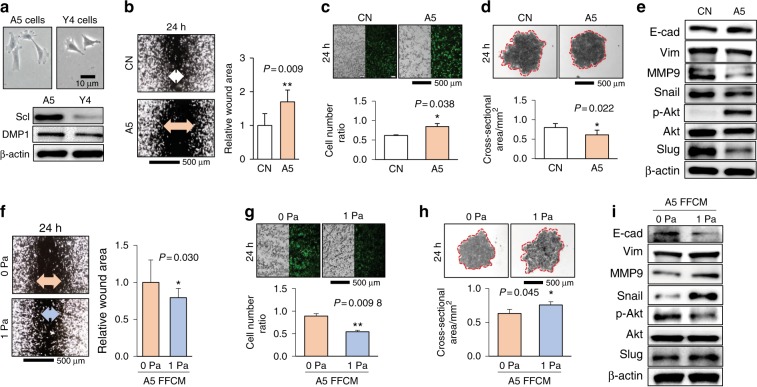
Differential effects of MLO-A5 osteocyte-derived conditioned medium (A5 CM) with and without fluid flow (FF) treatment at 1 Pa on TMD cells. CN control and A5 A5 CM. The single and double asterisks indicate *P* < 0.05 and *P* < 0.01, respectively. **a** Representative images of A5 and Y4 cells and the expression of Sclerostin (Scl) and DMP1. **b** Inhibition of 2-dimensional cellular migration in a scratch assay by A5 CM. **c** Stimulation of cell proliferation by A5 CM in an EdU assay. **d** Compaction of TMD tumor spheroids by A5 CM. **e** Downregulation of Vimentin (Vim), MMP9, Snail, and Slug and upregulation of E-cadherin (E-cad) and p-Akt by A5 CM. **f** Promotion of cellular migration in a scratch assay by A5 FFCM. **g** Reduction in cellular proliferation by A5 FFCM. **h** Expansion of TMD tumor spheroids by A5 FFCM. **i** Upregulation of Vimentin, MMP9, Snail, and Slug and downregulation of E-cadherin and p-Akt by A5 FFCM

We first conducted in vitro analysis using osteocyte-derived conditioned media with and without fluid flow treatment (Supplementary Fig. S1). In this study, we advanced the differentiation stage of parental MLO-A5 early-stage osteocytes by incubating them with ascorbic acid. A5 cells expressed a higher level of Sclerostin and DMP1, two osteocyte markers, compared with that of Y4 osteocyte-like cells (Fig. 1a). Using TMD breast cancer cells (a variant of MDA-MB-231 cells), we examined the effects of A5 CM and FFCM. A5 CM inhibited the healing of wounds in the scratch assay (Fig. 1b), stimulated cellular proliferation in an EdU assay (Fig. 1c), and compacted tumor spheroids (Fig. 1d). A5 CM also reduced Snail, Vimentin, MMP9 and Slug and elevated E-cadherin and p-Akt (Fig. 1e). Notably, A5 FFCM (1 Pa flow application) reversed the responses to A5 CM. FFCM at 1 Pa promoted cellular migration (Fig. 1f), reduced cellular proliferation (Fig. 1g), and expanded tumor spheroids (Fig. 1h). FFCM also upregulated Snail, Vimentin, MMP9 and Slug and downregulated E-cadherin and p-Akt (Fig. 1i).

### Migratory behaviors in response to A5 CM and FFCM

**Fig. 2:**
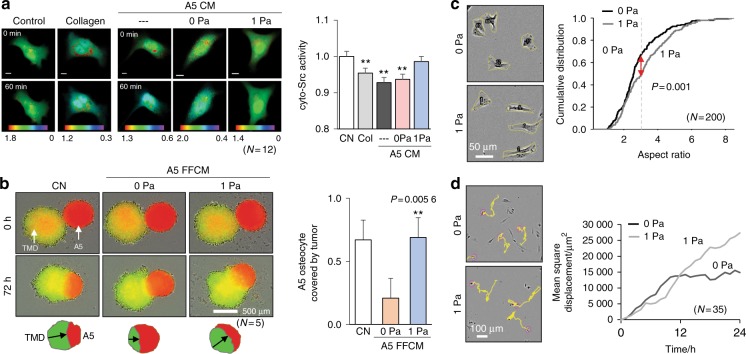
Visualization of FRET-based Src activity, tumor-osteocyte spheroid interactions, and migration of individual TMD cells. The double and triple asterisks indicate *P* < 0.01 and *P* < 0.001, respectively. **a** Representative images and quantification of FRET-based Src activity. **b** Interaction of TMD tumor spheroids (yellow) with A5 osteocyte spheroids (red) in 72 h. The tumor area (in %) is defined as the ratio of tumor spheroid area to the combined spheroid area. **c** Representative images of TMD cells in A5 CM and FFCM and the cumulative distribution of the cellular aspect ratio. **d** Representative trajectories of TMD cells in A5 CM and FFCM and the change in the mean-square displacement at 24 h

To further evaluate the differential effects of CM and FFCM at 1 Pa, we examined the activity level of Src using a FRET-based biosensor. Collagen treatment was used as a control to suppress Src based on our previous work.^9^ Src activity was downregulated by A5 CM as well as by incubation with 10 μg·mL^−1^ collagen, but the observed downregulation was reversed by FFCM (Fig. 2a). The migratory behaviors of three cell lines (TMD, MDA-MB-231, and 4T1 breast cancer cells) were also suppressed by A5 CM and Y4 CM (Supplementary Fig. S2a–d). The degree of suppression was stronger in the presence of A5 CM than Y4 CM.

Tumor spheroids presented high affinity to A5 spheroids in the control medium (Fig. 2b). A5 CM inhibited the migration of tumor spheroids (green) toward A5 spheroids (red), and this inhibition was suppressed by FFCM at 1 Pa. Live cell imaging revealed that individual tumor cells in FFCM appeared to be more slender with a higher aspect ratio (the ratio between the major and minor axes) than those in A5 CM (Fig. 2c). Furthermore, the trajectory of individual cells in the FFCM suggested a random walk in which the mean-square displacement was proportional to time, while the trajectory in the A5 CM showed a restricted random walk (Fig. 2d). Taken together, these cellular images also support the notion that FFCM at 1 Pa activated Src and induced tumor cell migration compared with that of A5 CM.

### Proteins differentially expressed in A5 CM and FFCM

**Fig. 3:**
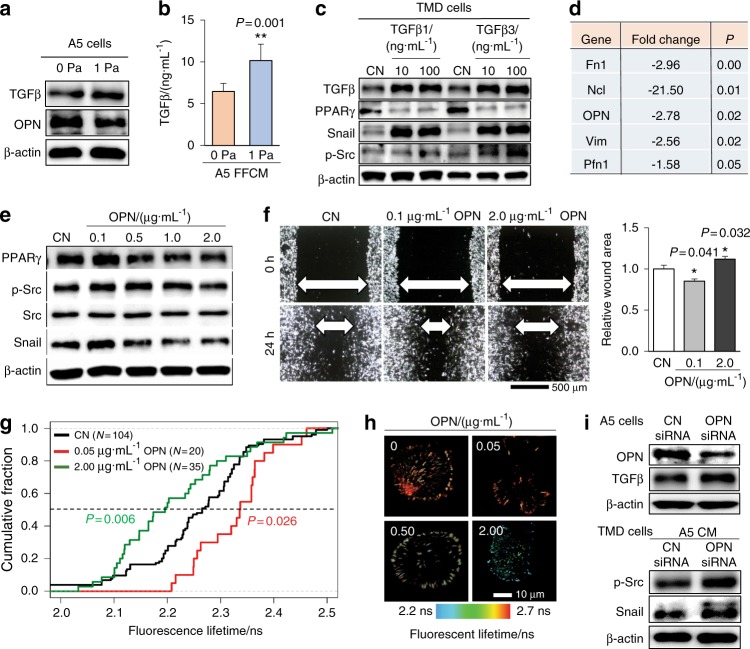
Effects of TGFβ and osteopontin on the expression of p-Src and Snail in TMD cells. CN control, Fn1 fibronectin, Ncl nucleolin, OPN osteopontin, Vim vimentin, and Pfn1 Profilin 1. The single and double asterisks indicate *P* < 0.05 and *P* < 0.01, respectively. **a** Expression of TGFβ and OPN in A5 cells with and without fluid flow. **b** ELISA-measured increase in TGFβ in A5 conditioned medium with fluid flow. **c** Expression of TGFβ, PPARγ, snail, and p-Src in response to TGFβ1 and TGFβ3. **d** Mass spectrometry-based detection of proteins that were differentially expressed in A5 CM and A5 FFCM. **e** Expression of PPARγ, p-Src and Snail in TMD cells in response to 0.1–2 μg·mL^−1^ OPN. **f** Alterations in 2-dimensional cellular migration of TMD cells in response to 0.1 and 2 μg·mL^−1^ OPN. **g** FRET-based fluorescent lifetime of the vinculin tension sensor in response to 0–2 μg·mL^−1^ OPN. **h** Color-coded fluorescence lifetime in response to 0, 0.05, 0.5, and 2 μg·mL^−1^ OPN. **i** OPN and TGFβ expression in OPN siRNA-treated A5 osteocytes and downregulation of p-Src and snail in TMD cells by OPN siRNA-treated A5 CM

We next evaluated the expression of TGFβ as a potential regulator of the action of A5 CM and FFCM, since TGFβ is known to play a vital role in osteolysis. Compared with the level in A5 CM, Western blot analysis and ELISA showed that TGFβin FFCM was increased (Fig. 3a, b). Snail and p-Src were increased in response to 10 and 100 ng·mL^−1^ TGFβ1 and β3, but PPARγ was decreased (Fig. 3c). To further investigate proteins that are potentially responsible for the actions of A5 CM and FFCM, we conducted mass spectrometry-based analysis. In addition to TGFβ, we identified five proteins that were differentially expressed (Fig. 3d). These proteins (Fibronectin, Nucleolin, Osteopontin, Vimentin, and Profilin 1) were more highly expressed in A5 CM than in FFCM. As predicted, treatment with each of these proteins, except for Profilin 1, downregulated Snail in TMD breast cancer cells and 4T1 mammary tumor cells (Supplementary Fig. S3). The level of Sclerostin was not altered (data not shown).

### Effects of OPN on cellular migration

Among the five predicted proteins, in vitro analysis showed that OPN significantly altered PPARγ, p-Src and Snail in a dose-dependent fashion (Fig. 3e, f). Notably, lower doses of OPN (0.1 μg·mL^−1^) upregulated PPARγ, p-Src and Snail, while higher doses (2 μg·mL^−1^) reduced them (Fig. 3e, f). Consistent with the suppression of p-Src and Snail, 2 μg·mL^−1^ OPN inhibited tumor cell wound healing. To further evaluate the role of OPN, we employed a FRET-based vinculin sensor and evaluated the molecular force of cell migration.^24^ This sensor was designed to alter the fluorescence lifetime under tensile force. We observed that a low dose of OPN (0.05 μg·mL^−1^) increased the fluorescence lifetime, indicating elevated tensile force, while a high dose of OPN (2 μg·mL^−1^) decreased it (Fig. 3g, h). Furthermore, partial silencing of OPN by siRNA in A5 osteocytes increased TGFβ, and CM downregulated p-Src and Snail in tumor cells (Fig. 3i). Collectively, these results were consistent with the dose-dependent role of OPN, in which fluid flow reduced OPN in A5 cells, induced tensile force on tumor cells, and stimulated migratory behaviors.

### Responses to fluid flow at 1 Pa and 0.25 Pa in tumor cell lines and primary breast cancer cells

**Fig. 4:**
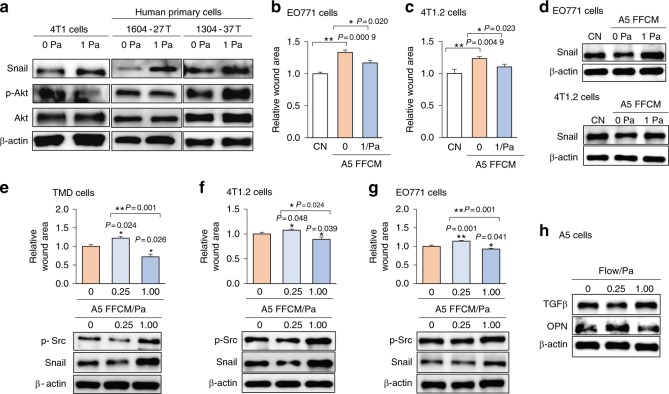
Responses to fluid flow in EO771, 4T1, 4T1.2, and primary human breast cancer cells. CN control. **a** Expression of Snail and p-Akt in 4T1 cells and two primary human breast cancer cells (1604-27 T and 1304-37 T) in response to A5 CM and FFCM. **b**, **c** Effect of A5 CM and FFCM on the migratory behavior of EO771 and 4T1.2 cells. **d** Expression of snail in response to A5 CM and FFCM in EO771 and 4T1.2 cells. **e–g** Dose-dependent effects of A5 FFCM (0.25 Pa and 1 Pa) in cellular migration and expression of p-Src and Snail in TMD cells, 4T1.2 cells, and EO771 cells. **h** Expression of TGFβ and OPN in response to 0.25 Pa and 1 Pa in MLO-A5 osteocytes

Thus far, the responses to CM and FFCM at 1 Pa were evaluated using a variant of MDA-MB-231 breast cancer cells. To further evaluate the responses to CM and FFCM, we employed other cell lines and primary human breast cancer cells. FFCM at 1 Pa upregulated Snail in 4T1 mammary tumor cells as well as in two types of primary breast cancer cells (Fig. 4a). EO771 and 4T1.2 mammary tumor cells had reduced wound healing in response to A5 CM (Fig. 4b, c). In these cell lines, Snail was downregulated by A5 CM and upregulated by FFCM at 1 Pa (Fig. 4d). Taken together, the responses to CM and FFCM were consistent among the six types of mammary tumor and breast cancer cells that we examined.

Using two levels of flow-induced shear stress (0.25 Pa and 1 Pa), we next examined whether fluid flow intensity alters the migratory behaviors or expression levels of flow-sensitive genes such as p-Src and Snail in TMD cells, 4T1.2 cells, and EO771 cells (Fig. 4e–g). When A5 osteocytes received low-level fluid flow (0.25 Pa), the tumor cell wound healing was suppressed, and p-Src and Snail were downregulated. In contrast, with 1 Pa shear stress, FFCM stimulated wound healing and elevated the levels of p-Src and Snail. In A5 osteocytes, the expression of TGFβ and OPN was also altered depending on the flow intensity (Fig. 4h). OPN was increased by 0.25 Pa and reduced by 1 Pa shear stress.

### Strain measurement

**Fig. 5:**
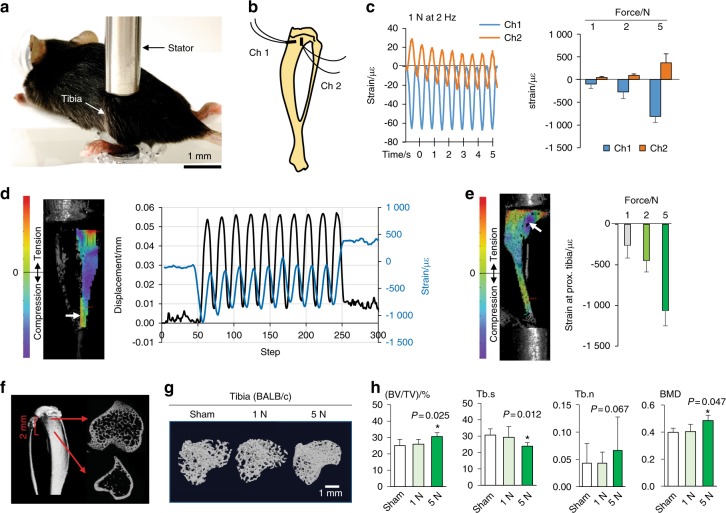
Tibia loading setup and strain measurement. **a** C57BL/6 mouse on a tibia loading device. **b**, **c** Strain measurement using a pair of strain gauges. **d** Representative dynamic measurement of displacement and strain using a digital image correlation (DIC) method. **e** DIC-based strain measurement of the proximal tibia in response to 1, 2, and 5 N loads to the tibia. The negative strain value is compressive in response to tibia loading. **f** Section of the proximal tibia (2 mm in length) analyzed by μCT. **g**, **h** Loading magnitude-dependent alterations in trabecular bone in the proximal tibia by tibia loading (1 N and 5 N) in BALB/c control mice without tumor inoculation (*N* = 6 per group)

In vitro analysis identified an antitumor action of osteocytes that is strengthened by FFCM at 0.25 Pa and reversed by FFCM at 1 Pa. To further evaluate the effect of mechanical stimulation, we employed a mouse model and conducted dynamic tibia loading (axial compression to the tibia) using different (low and medium) loads (Fig. 5a).

Prior to evaluating load-driven effects on tumor-induced osteolysis, we conducted strain measurements and evaluated the mechanical microenvironment in the proximal tibia in which tumor cells were inoculated. The first method, using a pair of strain gauges immobilized in the proximal tibia (Fig. 5b), detected load magnitude-dependent strain in C57BL/6 mice (Fig. 5c; *N* = 5). In response to 1 N loads at 2 Hz, for instance, the induced strain was 98 μstrains (peak compression at Channel 1) and 46 μstrains (peak tension at Channel 2). The second method using 3D digital correlation imaging (DCI) also showed that cyclic compression (2 Hz with 1, 2, and 5 N loads) induced load-dependent bone deformation and strain (Fig. 5d). The maximum compressive strain in the proximal tibia with DCI was 260 μstrains (1 N), 480 μstrains (2 N), and 1 060 μstrains (5 N) (Fig. 5e; *N* = 3). The observed variations in the strain values indicate a complex strain pattern in the proximal tibia in response to tibia loading. Using normal control BALB/c mice without tumor inoculation, we also evaluated the effect of daily tibia loading for 2 weeks on trabecular bone in the proximal tibia (Fig. 5f–h). The results showed that tibia loading at 5 N elevated BV/TV (bone volume ratio) and BMD (bone mineral density) and reduced Tb.s (trabecular separation). However, tibial loading at 1 N did not alter these parameters.

### Load-dependent effects of tibia loading

**Fig. 6:**
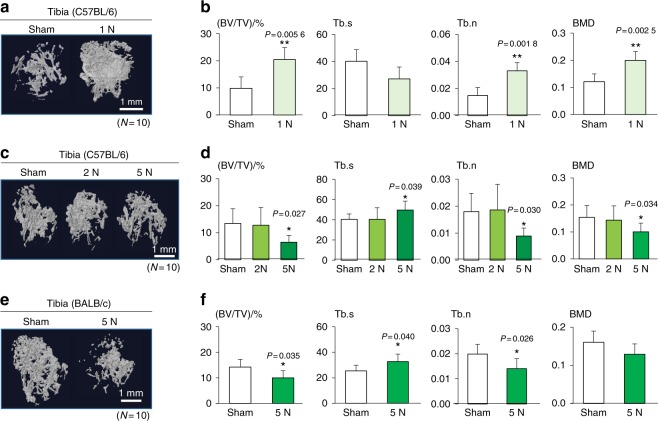
MicroCT-based evaluation of loading of the proximal tibia of C57BL/6 mice and BALB/c mice. BV/TV bone volume ratio normalized by total volume, Tb.s trabecular separation, Tb.n trabecular number, and BMD bone mineral density. The single and double asterisks indicate *P* < 0.05 and *P* < 0.01, respectively. **a**, **b** Beneficial effects of tibia loading (1 N) on the proximal tibiae of C57BL/6 mice (*N* = 10 per group). **c**, **d** Detrimental effects of tibia loading (2 and 5 N) on the proximal tibiae of C57BL/6 mice (*N* = 10 per group). **e**, **f** Detrimental effects of tibia loading (5 N) on the proximal tibiae of BALB/c mice (*N* = 10 per group)

**Fig. 7:**
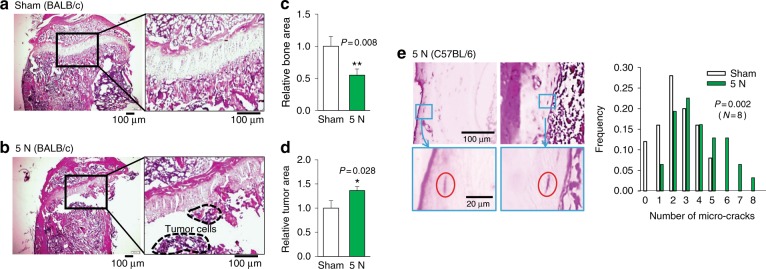
Histological analysis of the proximal tibiae and distal femurs of BALB/c mice in response to tibia loading (5 N). **a**, **b** Sagittal section of the tibia of the sham-control sample and tibia-loaded sample. The tibia-loaded samples presented more osteolytic lesions than those of the sham-control samples. **c**, **d** Quantification of bone area and tumor area in H&E-stained tibia sections. The area of the 5-N group is normalized to that of the sham-loading group. **e** Increase in the number of microcracks in cortical bone of the tibia-loaded samples

After intratibial injection of EO771 or 4T1.2 cells, we applied tibia loading to C57BL/6 or BALB/c mice at three intensities (1, 2, and 5 N loads). We observed that daily tibia loading of C57BL/6 mice with 1 N loads reduced the degradation of trabecular bone in the proximal tibia (Fig. 6a). More specifically, a significant increase in BV/TV, Tb.n (trabecular number), and BMD was observed (Fig. 6b). In contrast, osteolysis of trabecular bone was stimulated by tibia loading of C57BL/6 mice with 5 N loads (Fig. 6c, d), as well as BALB/c mice with intratibial injection of 4T1.2 cells and 5 N loads (Fig. 6e, f). No significant effect was observed in C57BL/6 mice with 2 N loads (Fig. 6c, d), and the administration of 1 N loads to BALB/c mice prevented bone loss in the tibia (Supplementary Fig. S4a, b). However, administration of 5 N loads to C57BL/6 mice damaged not only the proximal tibia but also the distal femur (Supplementary Fig. S4c, d). Of note, 5 N loads did not significantly damage the distal femur of BALB/c mice (data not shown). Histological images with H&E staining showed that the sample with 5 N loads presented significant bone damage distal to the growth plate in the proximal tibia in BALB/c mice compared with that of the sham loading sample (Fig. 7a, b). In the proximal tibia, loading at 5 N reduced the bone area ratio and increased the tumor area ratio (Fig. 7c, d). Fuchsin staining for microdamages indicated an increased number of microcracks in the proximal cortical bone in the tibia of the tumor-induced osteolytic samples with 5 N loads (Fig. 7e).

### Involvement of the mevalonate pathway in urine-derived VOCs

**Fig. 8:**
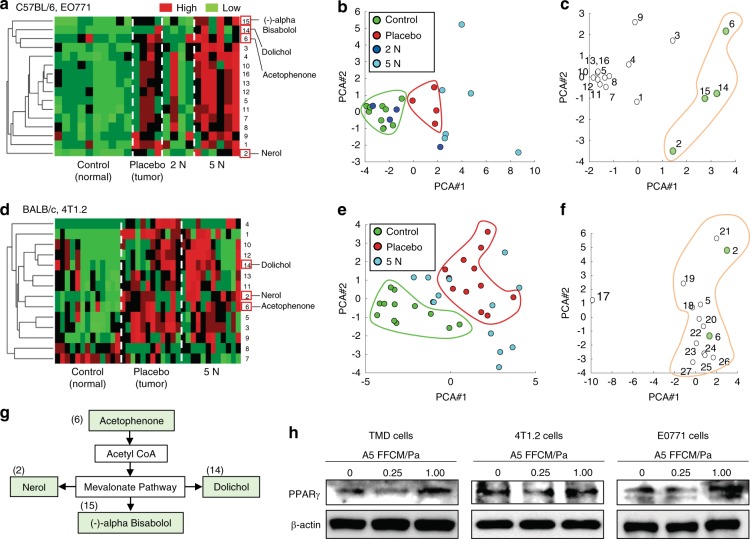
Analysis of urine-derived volatile organic compounds (VOCs) in C57BL/6 mice and BALB/c mice. **a**, **b** Hierarchical cluster analysis and principal component analysis of the urine derived from C57BL/6 mice (*N* = 4–10 per group). **c** Plots of VOCs in the principal component plane for C57BL/6 mice. **d**, **e** Hierarchical cluster analysis and principal component analysis of the urine derived from BALB/c mice (*N* = 12–13 per group). **f** Plots of VOCs in the principal component plane for BALB/c mice. **g** Four VOCs that were enriched in the tumor samples are linked to a mevalonate pathway. **h** Expression of PPARγ in TMD, 4T1.2, and EO771 tumor cells in response to A5 FFCM at 0.25 Pa and 1 Pa

We previously determined that terpenes and terpenoids in the mevalonate pathway are some of the VOCs that are significantly altered in mice with breast cancer tumors.^23^ To evaluate metabolic changes caused by loading, we analyzed urine-derived VOCs (Supplementary Table S1). Hierarchical clustering and principal component analysis (PCA) were conducted on samples from C57BL/6 and BALB/c mice (Fig. 8a–f). In the PCA plane, the normal control and the placebo control were separately positioned along the first PCA axis for both mouse breeds (Fig. 8b, e). The 2 N-loaded samples were positioned between the normal and placebo controls, while the 5 N-loaded samples were located away from the normal control. PCA of VOCs showed clustering of compounds along the first PCA axis, with the right cluster corresponding to the tumor-enriched VOCs (Fig. 8c, f). Among the tumor-enriched compounds, four VOCs (nerol, acetophenone, dolichol, and bisabolol) were linked to the mevalonate pathway (Fig. 8g). Because this pathway regulates lipid metabolism, we analyzed the expression levels of PPARγ, a transcription factor that is involved in lipid metabolism, in three tumor cell lines (TMD, 4T1.2, and EO771). In all cell lines, PPARγ was reduced by FFCM at 0.25 Pa and increased by FFCM at 1 Pa (Fig. 8h). Since the mevalonate pathway leads to cholesterol synthesis, we evaluated the effects of cholesterol in TMD and 4T1.2 cells. The results revealed that in both cell lines, cholesterol increased MMT-based cellular viability and migratory capability and upregulated MMP9, p-Src, and Snail (Supplementary Fig. S5).

### Load-dependent regulation of serum TGFβ, D-dimer, and cholesterol and gene expression

**Fig. 9:**
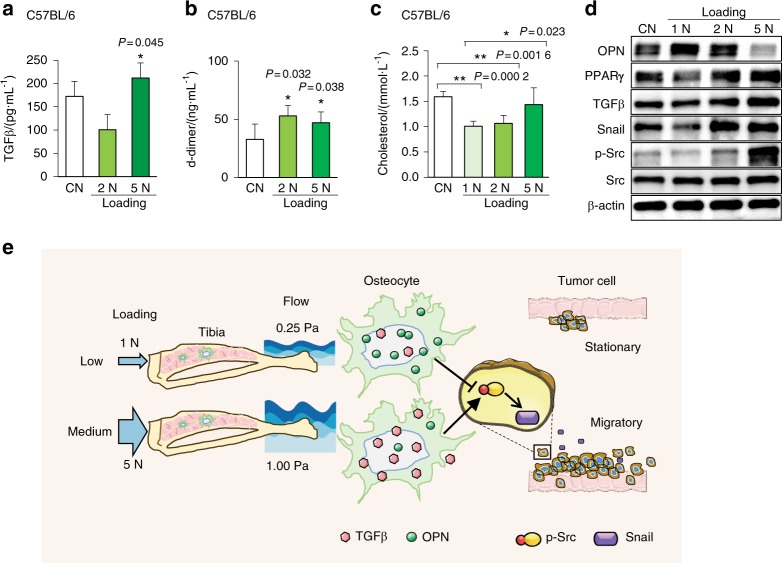
Loading effects on serum markers and tumor-induced muscle swelling. The single and double asterisks indicate *P* < 0.05 and *P* < 0.01, respectively. **a** Serum level of TGFβ. **b** Serum level of D-dimer, a marker of thrombosis. **c** Serum level of cholesterol. **d** Expression of PPARγ, OPN, TGFβ, Snail, p-Src, and Src in bone in response to tibia loading. **e** Proposed mechanism of osteocyte-tumor interactions. Osteocytes without mechanical stimulation suppress tumor migration by downregulating Src and Snail. Low-level mechanical loading (1 N) elevates OPN and further inhibits tumor migration, while medium-level loading (5 N) reduces OPN and stimulates tumor migration

In an attempt to associate fluid flow-driven gene regulation in vitro with load-driven responses in vivo, we analyzed the levels of selected markers in serum and the expression of metabolic and EMT-linked genes in bone marrow. ELISA results showed that 5 N loads increased TGFβ and D-dimer in the serum (Fig. 9a, b). Of note, TGFβ was elevated by FFCM at 1 Pa, and D-dimer is a marker for thrombus, indicating pathological loading of osteolytic bone. Because the mevalonate pathway leads to cholesterol production, we analyzed serum cholesterol levels in C57BL/6 mice. Loads at 1 N reduced the cholesterol level, but 5 N loads increased it (Fig. 9c). Western blot analysis of bone marrow-derived cells revealed that the level of OPN was increased by 1 N and reduced by 5 N loads. Conversely, the levels of PPARγ, TGFβ, Snail, and p-Src were reduced by 1 N and increased by 5 N loads (Fig. 9d). Collectively, these results suggest that medium-level mechanical stimulation (1 Pa shear stress in vitro and 5 N loads in vivo) promotes EMT-linked genes such as TGFβ, Snail, and p-Src and low-level stimulation (0.25 Pa shear stress in vitro and 1 N loads in vivo) inhibits them.

## Discussion

The main goal of this study was to determine whether osteocytes cultured under static conditions or mechanical stimulation express and/or release factors that alter the metastatic behaviors of breast cancer cells. Both in vitro and in vivo results demonstrated that osteocyte action on cancer cells differs with and without mechanical stimulation in an intensity-dependent manner. In vitro analysis revealed that A5 CM without FF inhibited cellular migration by downregulating Src activity and Snail expression. FFCM at 0.25 Pa further downregulated the Src-Snail axis. With FF at 1 Pa, however, the responses were altered. FFCM at 1 Pa upregulated Src and Snail and promoted cellular migration. Our findings on cellular migration at 1 Pa were consistent with previous work,^25^ and we identified further dependence of this effect on mechanical intensity. The FF-driven responses at 1 Pa were largely consistent with the EMT. Collectively, the present in vitro analysis indicates that FF at 1 Pa may alter the bone microenvironment from a pro-MET to pro-EMT milieu, while FF at 0.25 Pa changes the microenvironment to a further pro-MET milieu.

Metastatic dependence on loading intensity was also observed in the mouse model of tibial osteolysis. Previous loading work used SCID immunocompromised mice with the MDA-MB-231 cell line,^26^ but we employed two immunocompetent strains (BALB/c and C57BL/6) with two tumor cell lines (4T1.2 and EO771). While ~4 N loads at 4 Hz with ~600 μstrains for 5 min are reported to be beneficial,^27^ we observed load intensity-dependent responses with 1, 2, and 5 N loads at 2 Hz for 5 min. Our findings showed that 1 N loads with ~300 μstrains protected bone from tumor-induced osteolysis and 5 N loads with ~1 000 μstrains increased microcracks and bone degradation. No clear difference was observed with 2 N loads with ~500 μstrains. While intensity-dependent responses were observed in both mouse strains, the protective effects with 1 N loads were more significant in C57BL/6 than in BALB/c mice. Furthermore, the observed dependence on load intensity in vivo was consistent with in vitro FF responses, in which shear stress at 0.25 Pa inhibited the migratory behaviors of tumor cells and stimulated them at 1 Pa. The observed dependence on loading intensities was consistent with the serum concentrations of TGFβ and cholesterol, as well as the protein levels of PPARγ, OPN, and TGFβ in bone marrow, which presented loading intensity-dependent expression profiles.

The involvement of mechanical factors in tumor progression has been previously reported.^27–29^ The stiffness of the ECM is known to alter the proliferation and survival of not only tumor cells but also stromal cells in the tumor microenvironment.^28^ Tumor-induced pressure is reported to promote tumor growth in metastatic prostate cancer.^29^ In laryngeal carcinoma cells, FF is reported to induce the EMT by enhancing cellular migration.^4^ In this study, we applied FF to osteocytes in vitro and compressive skeletal loading to the tibia in vivo. Osteocytes in vivo receive oscillatory shear stress by load-driven deformation of the bone matrix and deformation-induced FF in the lacuno-canalicular network.^30^ In the present study, mechanical stimulation with 0.25 Pa FF and 1 N loads were beneficial, while stimulation with 1 Pa FF and 5 N loads were detrimental. These mechanical conditions heavily depend on culture conditions such as the serum concentration in the culture media as well as loading modalities and mouse strains used for skeletal loading. Further analysis is recommended to evaluate loading intensity-dependent tumor-osteocyte communication.

Mass spectrometry-based protein analysis and Western blotting revealed that Fibronectin, Nucleolin, Vimentin, and OPN were potential molecular contributors to the observed differences between A5 CM and FFCM. Fibronectin is a high molecular weight glycoprotein that binds to integrins.^31^ Nucleolin is known to be involved in cell differentiation, adhesion, inflammation and tumor development,^32^ while Vimentin is an intermediate filament protein that is used as a marker of mesenchymal-derived cells.^33^ In this study, we focused on the role of OPN in the responses to mechanical stimulation, since OPN is known to be involved in tumor progression.^19^

A schematic diagram of the load-driven action of OPN and TGFβ in osteocytes is illustrated, together with the subsequent expression of p-Src and Snail in tumor cells (Fig. 9e). The level of TGFβ in osteocytes was altered by OPN in a dose-dependent fashion. Tumor cells also responded to OPN in a manner that was dependent on its concentration in osteocytes. We observed that the level of OPN was differentially regulated depending on the intensities of FF on osteocytes and tibia loading. A low dose of OPN in osteocytes elevated p-Src and Snail in tumor cells, while high doses of OPN inhibited these factors. Of note, PPARγ was downregulated under 0.25 Pa FF and upregulated under 1 Pa FF. TGFβ suppresses PPARγ in human fetal lung fibroblasts,^34^ and our data also showed downregulation of PPARγ by TGFβ. Thus, unlike the regulation of p-Src and Snail, PPARγ was mainly regulated by OPN but not TGFβ.

OPN is a negatively charged, secreted phosphoprotein that is rich in the ECM of bone matrix. It has been reported that a secretory isoform of OPN induces the EMT, while its intracellular/nuclear isoform induces the MET.^35^ It has also been reported that cancer cells express different splice variants.^36^ While OPN may facilitate cellular adhesion and contribute to the migration and invasion of tumor cells,^37,38^ it is also possible that an excessive amount of OPN inhibits cellular migration because of strong tumor cell attachment to the substrate surface. The observed dose-dependent effect of OPN and the intensity-dependent responses to mechanical stimulation present a complex interplay of mechanosensitive ECM proteins in tumor-osteocyte communication.

In addition to characterizing loading effects at the loaded site, we analyzed load-driven metabolic responses in mouse urine.^23^ The beneficial effects of 1 N loads on the tibia at the loading site, as well as the detrimental in situ effects of 5 N loads, were associated with systemic changes in metabolites. Principal component analysis revealed that the tumor samples and 5 N-loaded samples were enriched for several volatile metabolites in the mevalonate pathway in urine-derived VOCs, while 2 N-loaded samples had reduced levels of these metabolites. The level of PPARγ in the tibial bone marrow was consistent with the changes in VOCs associated with lipid metabolism. The results of this study are consistent with the potential involvement of cholesterol in tumor progression,^22^ but the available data are preliminary. Further analysis is needed to examine the possibility of using VOCs to evaluate the efficacy of therapeutic interventions.

In this study, we mainly examined the regulation of the Snail-Src axis in EMT/MET transitions. Src is a nonreceptor tyrosine kinase, and many lines of evidence show its critical role in the progression of breast cancer.^39^ Src is activated by mechanical stimulation through ECM-integrin interactions.^40^ We observed that FFCM altered the expression and activity of Src in tumor cells that did not receive direct mechanical stimulation. FF at 1 Pa activated Src, while FF at 0.25 Pa inactivated Src. Growth factors can activate Src,^41^ and we observed that TGFβ1 was upregulated by FFCM at 1 Pa and downregulated by FFCM at 0.25 Pa. Snail expression was also altered by FFCM in a shear stress intensity-dependent manner.

This work demonstrates the involvement of mechanical stimulation in the regulation of the bone microenvironment and how tumor cells interact with the microenvironment. While the presented results reveal a novel feature of tumor-osteocyte interactions, the study has a few limitations. Our experiments used mouse bone cells and human cancer cells, and the potential effects of cross-species interactions should be taken into consideration. Interactions might be age-dependent. Furthermore, the bone microenvironment includes many other types of cells, such as osteoclasts, osteoblasts, and immune cells, which may respond differently to FF. The role of Wnt signaling and the potential involvement of Lrp5/Lrp6 coreceptors should be investigated in connection with the mechanotransduction of bone.^42^

In summary, this study revealed that osteocyte-tumor communication in the presence or absence of mechanical stimulation induced a substantially different effect on tumor cell behaviors. OPN, identified by mass spectrometry, was differentially expressed in A5 CM and FFCM, as well as in bone marrow from loaded and nonloaded tibiae, and was at least in part responsible for the observed responses in the Src-Snail regulatory axis in tumor cells. The results of tumor-osteocyte signaling suggest that strategies to prevent bone metastasis associated with breast cancer might benefit from the inclusion of mechanical loading of the skeleton, but the loading intensity should be carefully monitored.

## Materials and methods

### Cell culture

Human breast cancer cell-derived MDA-MB-231 cells, TMD cells (CH3 BioSystems, Amherst, NY, USA), 4T1 cells, 4T1.2 mammary tumor cells (obtained from Dr R. Anderson at the Peter MacCallum Cancer Institute, Australia), and EO771 mammary tumor cells were grown in DMEM (Corning, Inc., Corning, NY, USA). MLO-A5 and MLO-Y4 osteocyte-like cells (obtained from Dr L. Bonewald at Indiana University, USA) were grown in αMEM (Gibco, Carlsbad, CA, USA). For the tumor cells, the culture media were supplemented with 10% fetal bovine serum (FBS) and antibiotics. For MLO-A5 cells, the media contained 5% FBS and 5% fetal calf serum. Primary breast cancer cells (1604-27 T and 1304-37 T) were cultured in a 3:1 v/v mixture of F-12 and DMEM supplemented with 5% FBS, 0.4 µg·mL^−1^ hydrocortisone, 5 µg·mL^−1^ insulin, 8.4 ng·mL^−1^ cholera toxin, 10 ng·mL^−1^ epidermal growth factor, 24 µg·mL^−1^ adenine, and 5 μmol·L^−1^ Y-27632. The cells were maintained at 37 °C with 5% CO_2_. Conditioned media (A5 CM and Y4 CM) were collected from cells at ~80% confluence after 24 h incubation in media consisting of 1% FBS and antibiotics.

### EdU and scratch assays

Cellular proliferation was examined using a fluorescence-based cell proliferation kit (Click-iT™ EdU Alexa Fluor™ 488 Imaging Kit; Thermo-Fisher, Waltham, MA, USA). After fluorescent labeling, we counted the number of fluorescently labeled cells and determined the ratio to the total number of cells. A wound-healing scratch assay was utilized to evaluate 2-dimensional cell motility. In brief, cells were grown on 12-well plates, and a plastic tip was used to scratch a gap onto the cell layer. After incubation, the areas that were newly occupied with cells in the scratched zone were imaged and measured with ImageJ (National Institutes of Health, Maryland, USA).

### Application of fluid flow

Using the previously described procedure,^43^ oscillatory fluid flow at 1 Hz was applied for 1 h to MLO-A5 cells in a parallel plate flow chamber. A5 FFCM was collected with shear stress at 0.25 Pa or 1 Pa, and A5 CM was obtained without applying fluid flow.

### Western blot analysis and ELISA

Cells were lysed in radioimmunoprecipitation assay (RIPA) buffer, and proteins were fractionated using 10% SDS gels. We used antibodies against Akt, p-Akt, E-cadherin, Slug, Snail, Src, p-Src, TGFβ, Vimentin (Cell Signaling, Danvers, MA, USA), OPN, MMP9, PPARγ (Santa Cruz Biotechnology, Dallas, Texas, USA) and β-actin (Sigma). Protein levels were assayed using a SuperSignal West Femto maximum sensitivity substrate (Thermo Fisher Scientific). Using ELISA kits, the levels of TGFβ1 (Thermo Fisher), D-dimer and OPN (MyBioSource, San Diego, CA, USA) in A5 CM and FFCM were determined.

### Spheroid assay, conditioned media exchange assay, and RNA interference

The cells were cultured in a U-bottom low-adhesion 96-well plate (S-Bio, Hudson, NH, USA). All spheroid assays were performed in complete αMEM (10% FBS, 1% antibiotics). To evaluate the effect of conditioned media, spheroids were formed in separate wells for 48 h, and the media was removed and replaced with conditioned media. RNA interference was conducted using siRNA specific to OPN (Cat #AM16708, Life Technologies) with a negative siRNA (Silencer Select #1, Life Technologies) as a nonspecific control using the previously described procedure.^44^

### Fluorescence resonance energy transfer (FRET)

To evaluate the role of Src in response to conditioned media, Src activity was quantified by FRET imaging as previously described.^20^ A Src-specific biosensor was labeled with cyan fluorescent protein (CFP) and yellow fluorescent protein (YFP). Time-lapse images were acquired at an interval of 5 min, and the emission ratio of YFP/CFP for individual cells was computed to determine the activity levels using NIS-Elements software (Nikon).

To evaluate the tension force at a focal adhesion in the presence of varying concentrations of OPN, a plasmid expressing a vinculin tension sensor was transfected, and the fluorescence lifetime images were acquired by a custom-made microscope built on a laser scanning confocal microscope (FluoView 1000, Olympus) using the previously described procedures.^21^ Of note, an increase in the tension force of the vinculin sensor indicated a decrease in FRET efficiency and an increase in fluorescence lifetime.

### Mass spectrometry-based protein identification

Three samples of A5 CM and FFCM each were harvested and freeze-dried. Using a previously described procedure,^9^ proteins in the freeze-dried samples were analyzed by reverse-phase HPLC-ESI-MS/MS with a Dionex-Thermo Fisher Scientific UltiMate 3000 RSLC nano System (Thermo Fisher Scientific) coupled to a Q-Exactive HF Hybrid Quadrupole Orbitrap MS (Thermo Fisher Scientific).

### Animal model

The experimental procedures were approved by the Indiana University Animal Care and Use Committee and were in compliance with the Guiding Principles in the Care and Use of Animals endorsed by the American Physiological Society. In the mouse model of osteolysis,^43,44^ 48 BALB/c and 50 C57BL/6 female mice (~6 weeks old, Envigo) received intratibial injections of 4T1.2 and EO771 cells (2.5 × 10^5^ cells in 20 μL PBS) in the left tibia, respectively. After two days of recovery, tibia loading was performed daily on the tumor-inoculated tibiae as previously described.^45^ Using an ElectroForce device (Bose), the left tibiae of the mask-anesthetized mice were given daily loads of 1 N, 2 N, or 5 N (peak-to-peak) at 2 Hz for 5 min. The animals were sacrificed on day 14. We harvested the tibiae and femurs for histology and μCT imaging, bone marrow-derived cells for Western blot analysis, urine for VOC analysis, and blood for ELISA.

### Strain measurement

Strain in the tibia in response to axial compressive loading was measured using a strain measurement unit (EDX-14A; Kyowa Americas Inc.), as well as 3D digital image correlation (3D-DIC). In the former method, a pair of strain gauges (SKF-27085, 200 μm gauge length; Kyowa) was immobilized to the proximal tibia in an orthogonal direction, and the tibia was compressed with 1 N, 2 N, or 5 N loads at 2 Hz. In the latter method, we employed the previously described procedure,^46^ and the strain was calculated from the dynamic pattern of displacements on the tibial surface.

### MicroCT imaging

Microcomputed tomography was performed using Skyscan 1172 (Bruker-MicroCT, Kontich Belgium). Scans were performed at a pixel size of 8.99 μm. Using manufacturer-provided software, the images were reconstructed (nRecon v1.6.9.18) and analyzed (CTan v1.13). We determined BV/TV (bone volume normalized to total volume), Tb.s (trabecular separation), Tb.n (trabecular number), and cortical BMD (bone mineral density) of the proximal tibial segment (2 mm thick) and distal femoral segment (1 mm thick) from the growth plate to the distal section and proximal section, respectively.

### Histology

Bone samples were fixed in 4% paraformaldehyde in PBS and decalcified in a 10% EDTA solution. They were then dehydrated through a series of graded alcohols, cleared in xylene, and embedded in paraffin. H&E staining was conducted on the sagittal sections, and the distribution of tumor cells in the tibial bone cavity was determined. Of note, the bone ratio was defined as the ratio of the bone area to the total area in the proximal tibia (1 mm below the growth plate), while the tumor ratio was defined as the ratio of the tumor area to the total area. Fuchsin staining was also performed to detect microdamage in the bone. The number of Fuchsin-stained microcracks in the cortical bone of the proximal tibia (2 mm from the growth plate) was counted in a blinded fashion in samples from the sham-loaded group and the tibia-loaded group at 5 N.

### Analysis of volatile organic compounds (VOCs)

Twenty-four urine samples (50 μL) were collected from five groups of C57BL/6 mice (10 from the normal control, 4 from placebo, 4 from 2 N-loaded mice, and 6 from 5 N-loaded mice). For BALB/c mice, 37 samples were collected (13 from normal controls, 12 from placebo, and 12 from 5 N-loaded mice). The normal control samples were collected on day −1, and the placebo and loaded samples were collected on day 15 (C57BL/6 mice) and day 19 (BALB/c mice). VOCs were analyzed by solid-phase microextraction coupled with gas chromatography-mass spectrometry with quadrupole time-of-flight using a previously described procedure.^23^ A matrix with 531 VOCs for C57BL/6 samples and a matrix with 581 VOCs for BALB/c mice were generated. Compounds that were statistically significant in the normal control and placebo groups by Student’s *t* test were used for hierarchical clustering analysis, and principal component analysis was performed using MATLAB (R2018b; Math Works, Natick, MA, USA). Compounds were identified by spectral reference to NIST14, and metabolic pathway analysis was carried out via the KEGG database.

### Statistical analysis

For cell-based experiments, three or four independent experiments were conducted, and the data are expressed as the mean ± S.D. CM or FFCM from different dishes were collected for each independent experiment. Treatment groups were compared with vehicle-treated controls and no-flow control conditions. For experiments using the animal model, the metastasis model was compared with vehicle-injected control, and mechanically loaded metastatic mice were compared with sham-loaded metastatic mice. Statistical significance was evaluated using one-way analysis of variance (ANOVA). Post hoc statistical comparisons with control groups were performed using Bonferroni correction with statistical significance set at *P* < 0.05. A nonparametric Kolmogorov–Smirnov test was applied to compare cell aspect ratios. The single and double asterisks in the figures indicate *P* < 0.05 and *P* < 0.01, respectively.

## Supplementary information

Supplementary information

## References

[1] Huang C, Ogawa R (2010). Mechanotransduction in bone repair and regeneration. FASEB J..

[2] Goldring SR (2015). The osteocyte: key player in regulating bone turnover. RMD Open.

[3] Huang Q (2018). Fluid shear stress and tumor metastasis. Am. J. Cancer Res..

[4] Liu S (2016). Fluid shear stress induces epithelial-mesenchymal transition (EMT) in Hep-2 cells. Oncotarget.

[5] Tse JM (2012). Mechanical compression drives cancer cells toward invasive phenotype. Proc. Natl Acad. Sci. USA.

[6] Chen YC, Sosnoski DM, Mastro AM (2010). Breast cancer metastasis to the bone: mechanisms of bone loss. Breast Cancer Res..

[7] Weilbaecher KN, Guise TA, McCauley LK (2011). Cancer to bone: a fatal attraction. Nat. Rev. Cancer.

[8] Bonecchi R, Locati M, Mantovani A (2011). Chemokines and cancer: a fatal attraction. Cancer Cell.

[9] Chen A (2018). Attraction and compaction of migratory breast cancer cells by bone matrix proteins through tumor-osteocyte interactions. Sci. Rep..

[10] Corsa C (2016). The action of discoidin domain receptor 2 in basal tumor cells and stromal cancer associated fibroblasts is critical for breast cancer metastasis. Cell Rep..

[11] Vogel W, Gish GD, Alves F, Pawson T (1997). The discoidin domain receptor tyrosine kinases are activated by collagen. Mol. Cell.

[12] Pichler K (2013). Expression of matrix metalloproteinases in human growth plate chondrocytes is enhanced at high levels of mechanical loading: a possible explanation for overuse injuries in children. Bone Jt. J..

[13] Chang SH (2019). Excessive mechanical loading promotes osteoarthritis through the gremlin-1-NF-kB pathway. Nature Commun..

[14] Patel JB (2011). Control of EVI-1 oncogene expression in metastatic breast cancer cells through microRNA miR-22. Oncogene.

[15] Kato Y (2001). Establishment of an osteoid preosteocyte-like cell MLO-A5 that spontaneously mineralizes in culture. J. Bone Min. Res..

[16] Chiechi A (2013). Role of TGF-*β* in breast cancer bone metastases. Adv. Biosci. Biotechnol..

[17] Ishizawar R, Parsons SJ (2004). C-Src and cooperating partners in human cancer. Cancer Cell.

[18] McCarthy N (2012). Signalling: SRC and survival. Nat. Rev. Cancer.

[19] Zhao H (2018). The role of osteopontin in the progression of solid organ tumour. Cell Death Dis..

[20] Wan Q (2017). Subcellular domain-dependent molecular hierarchy of Src and FAK in mechanotransduction and cytokine signaling. Sci. Rep..

[21] Li F (2019). Vinculin force sensor detects tumor-osteocyte interactions. Sci. Rep..

[22] Kuzu OF (2016). The role of cholesterol in cancer. Cancer Res.

[23] Woollam M (2019). Detection of volatile organic compounds (VOCs) in urine via gas chromatography-mass spectrometry QTOF to differentiate between localized and metastatic models of breast cancer. Sci. Rep..

[24] Grashoff C (2010). Measuring mechanical tension across vinculin reveals regulation of focal adhesion dynamics. Nature.

[25] Ma Y (2018). Mechanical regulation of breast cancer migration and apoptosis via direct and indirect osteocyte signaling. J. Cell. Biochem..

[26] Lynch ME (2013). In vivo tibial compression decreases osteolysis and tumor formation in a human metastatic breast cancer model. J. Bone Min. Res..

[27] Menon S, Beningo K (2011). Cancer cell invasion is enhanced by applied mechanical stimulation. PLoS ONE..

[28] Ishihara S, Inman DR, Li WJ, Ponik SM, Keely PJ (2017). Mechano-signal transduction in mesenchymal stem cells induces prosaposin secretion to drive the proliferation of breast cancer cells. Cancer Res.

[29] Sottnik JL, Dai J, Zhang H, Campbell B, Keller ET (2015). Tumor-induced pressure in the bone microenvironment causes osteocytes to promote the growth of prostate cancer bone metastasis. Cancer Res.

[30] Fritton SP, Weinbaum S (2009). Fluid and solute transport in bone: flow induced mechanotransduction. Annu Rev. Fluid Mech..

[31] Danen EH, Yamada KM (2001). Fibronectin, integrins, and growth control. J. Cell Physiol..

[32] Palmieria D (2015). Human anti-nucleolin recombinant immunoagent for cancer therapy. Proc. Natl Acad. Sci. USA.

[33] Satelli A, Li S (2011). Vimentin in cancer and its potential as a molecular target for cancer therapy. Cell Mol. Life Sci..

[34] Lakshmi (2017). Transforming growth factor β suppresses peroxisome proliferator activated receptor γ expression via both SMAD binding and novel TGFβ inhibitory elements. Biochemical J..

[35] Jia R (2016). Osteopontin facilitates tumor metastasis by regulating epithelial-mesenchymal plasticity. Cell Death Dis..

[36] He B, Mirza M, Weber G (2006). An osteopontin splice variant induces anchorage independence in human breast cancer cell. Oncogene.

[37] Wai P, Kuo P (2008). Osteopontin: regulation in tumor metastasis. Cancer Metastasis Rev..

[38] Wei R, Wong J, Kwok H (2017). Osteopontin– a promising biomarker for cancer therapy. J. Cancer.

[39] Myoui A (2003). C-SRC tyrosine kinase activity is associated with tumor colonization in bone and lung in an animal model of human breast cancer metastasis. Cancer Res..

[40] Courter DL, Lomas L, Scatena M, Giachelli CM (2005). Src kinase activity is required for integrin aVb3 mediated activation of nuclear factor kB. J. Biol. Chem..

[41] Mon NN, Senga T, Ito S (2017). Interleukin 1b activates focal adhesion kinase and Src to induce matrix metalloproteinase 9 production and invasion of MCF-7 breast cancer cells. Oncol. Lett..

[42] Kang KS, Robling AG (2014). New insights into Wnt-Lrp5/6-b-catenin signaling in mechanotransduction. Frot Endocrinol..

[43] Liu D (2008). Activation of extracellular signal regulated kinase (ERK1/2) by fluid shear is Ca2+ and ATP dependent in MC3T3-E1 osteoblasts. Bone.

[44] Liu S (2018). Osteocyte-driven downregulation of snail restrains effects of Drd2 inhibitors on mammary tumor cells. Cancer Res..

[45] Dodge T (2012). Mechanical loading, damping, and load-driven bone formation in mouse tibiae. Bone.

[46] Li J, Yang G, Siebert T, Shi MF, Yang L (2018). A method of the direct measurement of the true stress-strain curve over a large strain range using multi-camera digital image correlation. Opt. Lasers Eng..

