# Supplementary material for: Skeletal loading regulates breast cancer-associated osteolysis in a loading intensity-dependent fashion
Source: Bone Res. 2020 Feb 14;8:9. doi: 10.1038/s41413-020-0083-6 (PMC7021802; doi:10.1038/s41413-020-0083-6)
Supplement: Supplementary file 1 — Supplementary information [file 41413_2020_83_MOESM1_ESM.pdf]

Supplementary Information

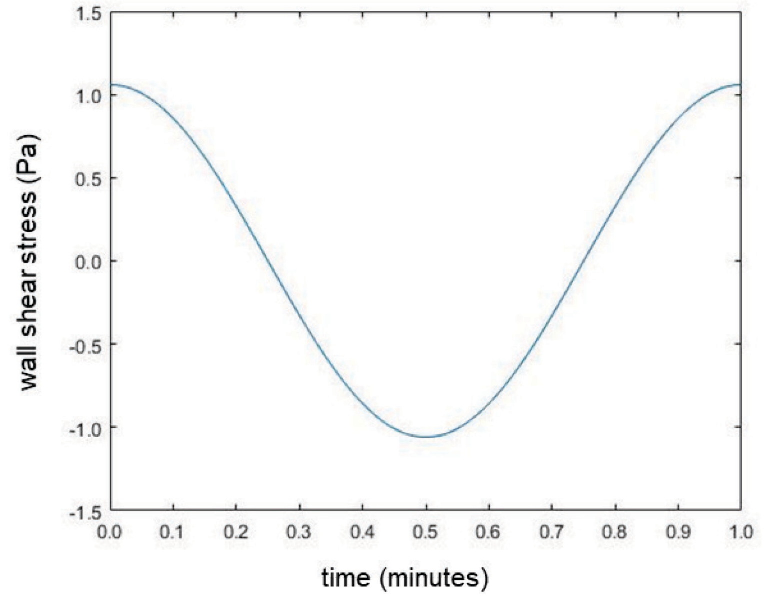

Supplementary Fig. S1. Estimation of wall shear stress in Pa in a sinusoidal flow in a pair of parallel plates (0.3 mm thickness) at 1 Hz with a bulk flow velocity of 5.6 cm/s, and kinematic viscosity of 0.72 × 10^{-6} m^2/s. The flow equation between the parallel plates, $\frac{1}{\nu}\frac{\partial w(t,y)}{\partial t} - \frac{\partial^2 w(t,y)}{\partial y^2} = \frac{\pi}{2\mu}(\nabla p)e^{i2\pi t}$, was analytically solved, in which $w(t,y)$ = velocity, ν = kinematic viscosity, μ = dynamic viscosity, and ∇p = pressure gradient. Wall shear stress was estimated as a product of dynamic velocity and velocity gradient.

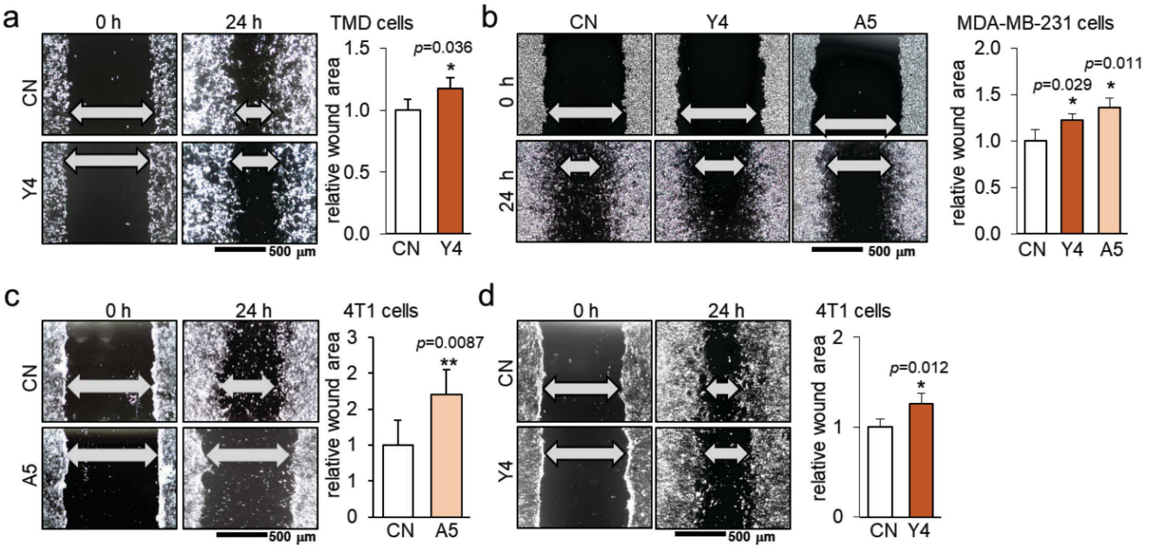

Supplementary Fig. S2. Comparison of MLO-A5 and MLO-Y4 cells. Of note, CN = control, A5 = A5 CM, and Y4 = Y4 CM. The single and double asterisks indicate $p < 0.05$ and $p < 0.01$, respectively. **a.** Suppression of TMD migration in response to Y4 CM. **b.** Suppression of MDA-MB-231 migration in response to A5 and Y4 CM. **c&d.** Suppression of 4T1 migration in response to A5 CM and Y4 CM.

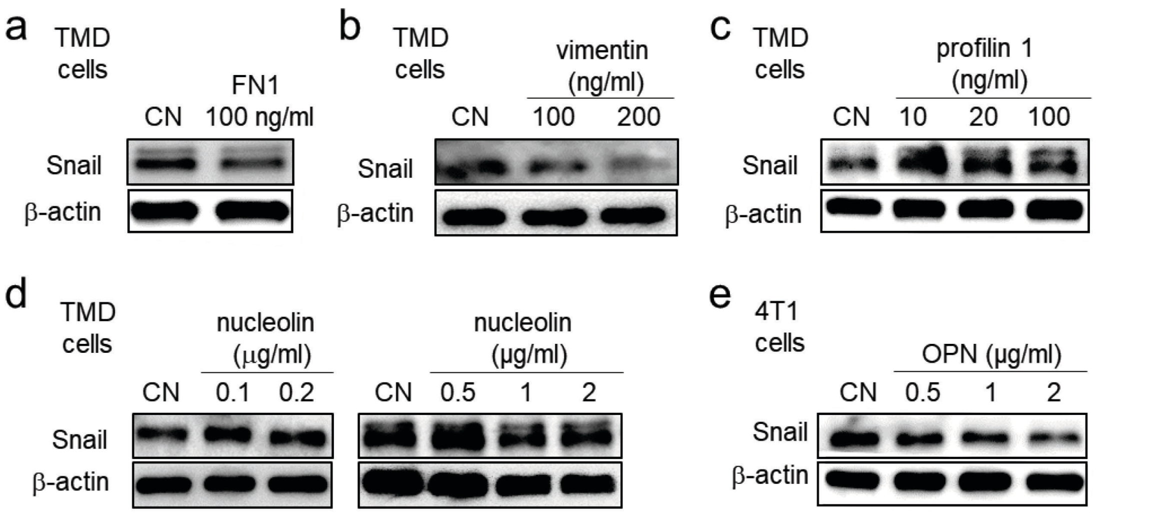

Supplementary Fig. S3. Effects of Fibronectin, Vimentin, Profilin 1, Nucleolin, and Osteopontin on the expression of Snail in TMD and 4T1 cells. Of note, CN = control, and FN1 = Fibronectin. **a-d.** Expression of p-Akt, p-Src, and snail in TMD cells in response to Fibronection, Vimentin, Profilin 1, and Nucleolin, respectively. **e.** Expression snail in 4T1 cells in response to osteopontin.

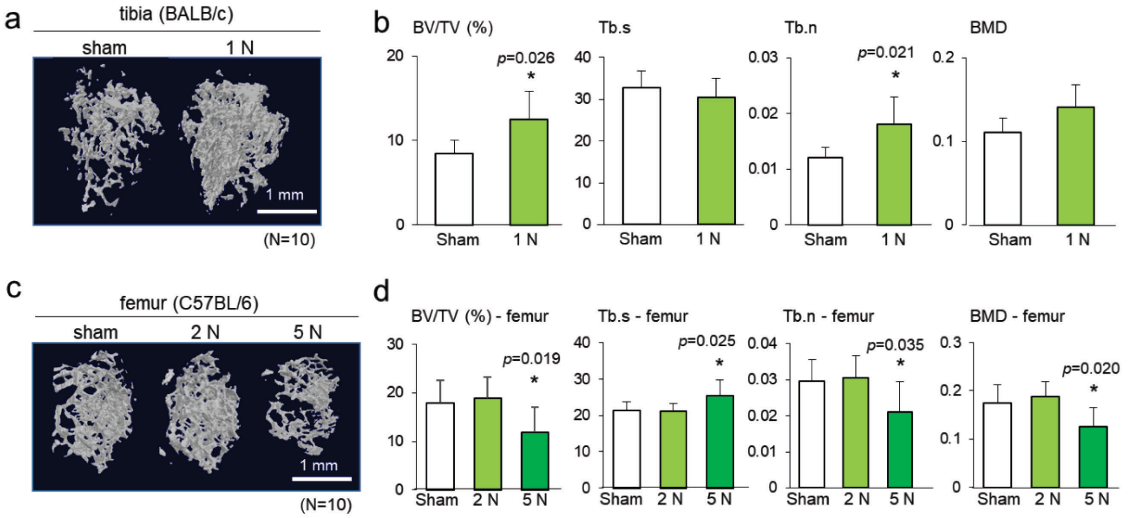

Supplementary Fig. S4. µCT-based evaluation of the proximal tibia and distal femur in response to tibia loading. Of note, BV/TV = bone volume ratio normalized by total volume, Tb.s = trabecular separation, Tb.n = trabecular number, and BMD = bone mineral density. The single asterisk indicates $p < 0.05$. **a&b.** Beneficial effects of tibia loading (1 N) to the proximal tibia of BALB/c mice (N=10/group). **c&d.** Detrimental effects of tibia loading (5 N) to the distal femur of C57BL/6 mice (N=10/group).

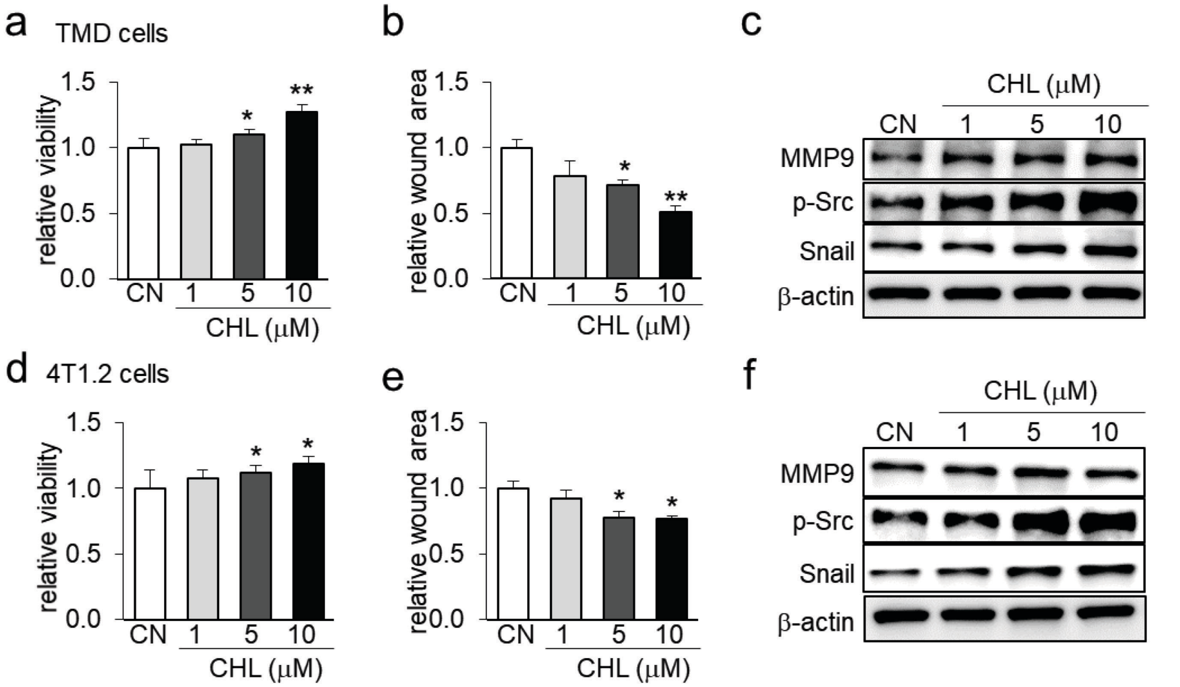

Supplementary Fig. S5. Effects of cholesterol in TMD and 4T1.2 cells. **a&b.** Effect of cholesterol reduction in cellular viability and migration of TMD cells. **c.** Expression of MMP9, p-Src, and Snail in TMD cells in response to cholesterol. **d&e.** Effect of cholesterol reduction in cellular viability and migration of 4T1.2 cells. **f.** Expression of MMP9, p-Src, and Snail in 4T1.2 cells in response to cholesterol.

.

Supplementary Table S1. List of volatile organic compounds (VOCs) derived from C57BL/6 and BALB/c mice.

Compound ID	IUPAC name	Compound ID	IUPAC name
1	1-octen-3-ol	15	(2S)-6-methyl-2-[(1S)-4-methylcyclohex-3-en-1-yl]hept-5-en-2-ol
2	cis-3,7-dimethylocta-2,6-dien-1-ol	16	2-methoxybenzene-1,4-diol
3	pent-2-en-4-ynyl furan-2-carboxylate	17	5-ethylcyclopentene-1-carboxylic acid
4	2-ethylhexan-1-ol	18	3-ethyl-3-phenylazetidine-2,4-dione
5	ethyl 4-ethoxybenzoate	19	methyl 2-methylprop-2-enoate
6	1-(2,5-dimethylphenyl)ethanone	20	(E)-4-oxohex-2-enal
7	2,3,5,5,8a-pentamethyl-4a,5,6,7,8,8a-hexahydro-4H-chromene	21	2,4,6-tri(propan-2-yl)phenol
8	3,5,5-trimethylcyclohex-2-en-1-one	22	pent-4-enyl propanoate
9	(Z)-non-3-en-1-ol	23	2-methoxy-4-methyl-1-pentylbenzene
10	undecan-2-one	24	1-methyl-4-(6-methylhepta-2,5-dien-2-yl)cyclohexene
11	3-tert-butyl-3,4-dihydro-2H-naphthalen-1-one	25	1-methoxy-4-(2-methylpropyl)benzene
12	2,4-ditert-butylphenol	26	(1S,5S,6R)-4,6-dimethyl-6-(4-methylpent-3-enyl) bicyclo[3.1.1]hept-3-ene
13	8,8,9-trimethyl-deca-3,5-2,7-dione	27	(4-fluorophenyl)methanol,2-methylpropyl ether
14	3-methyl-2-buten-1-ol		
